# Spinal cord injury increases the reactivity of rat tail artery to angiotensin II

**DOI:** 10.3389/fnins.2014.00435

**Published:** 2015-01-06

**Authors:** Hussain Al Dera, James A. Brock

**Affiliations:** ^1^Department of Anatomy and Neuroscience, University of MelbourneMelbourne, VIC, Australia; ^2^Basic Medical Sciences, College of Medicine, King Saud bin Abdulaziz University for Health SciencesRiyadh, Saudi Arabia

**Keywords:** spinal cord injury, neurovascular transmission, sympathetic nerves, angiotensin II, vascular reactivity

## Abstract

Studies in individuals with spinal cord injury (SCI) suggest the vasculature is hyperreactive to angiotensin II (Ang II). In the present study, the effects of SCI on the reactivity of the rat tail and mesenteric arteries to Ang II have been investigated. In addition, the effects of SCI on the facilitatory action of Ang II on nerve-evoked contractions of these vessels were determined. Isometric contractions of artery segments from T11 (tail artery) or T4 (mesenteric arteries) spinal cord-transected rats and sham-operated rats were compared 6–7 weeks postoperatively. In both tail and mesenteric arteries, SCI increased nerve-evoked contractions. In tail arteries, SCI also greatly increased Ang II-evoked contractions and the facilitatory effect of Ang II on nerve-evoked contractions. By contrast, SCI did not detectably change the responses of mesenteric arteries to Ang II. These findings provide the first direct evidence that SCI increases the reactivity of arterial vessels to Ang II. In addition, in tail artery, the findings indicate that Ang II may contribute to modifying their responses following SCI.

## Introduction

Angiotensin II (Ang II) plays an important role in the regulation of peripheral resistance. Although acutely interrupting Ang II signaling produces at most a small reduction in blood pressure in healthy humans, Ang II does contribute to the elevation of blood pressure that occurs when the activity of the renin-angiotensin system is increased (Timmermans et al., 1993). Following spinal cord injury (SCI), when supraspinal regulation of the vasculature below the lesion is lost, the renin-angiotensin system also plays a greater role in regulating vascular resistance (Popa et al., 2010). In comparison with able-bodied subjects, individuals with thoracic or cervical SCI have higher renin levels in their plasma when supine (Mathias et al., 1980; Kooner et al., 1988; Groothuis et al., 2010b). Furthermore, in patients with cervical SCI, there was a rapid and marked rise in plasma renin levels during a head up tilt (Mathias et al., 1980). This posturally evoked renin release occurs independently of sympathetic nerve activity and most likely depends on the fall in renal perfusion pressure causing stimulation of juxtaglomerular renin secreting cells (see Mathias et al., 1980).

Ang II mediates its excitatory effects on arterial vessels both by directly activating the vascular muscle and by augmenting sympathetic nerve-mediated constriction (Nap et al., 2003). There is indirect evidence that the vasculature of SCI patients is hyperreactive to Ang II (Krum et al., 1992; Groothuis et al., 2010b). Furthermore, treatment with the angiotensin converting enzyme inhibitor captopril is reported to lower blood pressure during spinal reflex-evoked hypertensive episodes [so called autonomic dysreflexia (AD)] that are triggered by bladder or colon distension or by other unheeded sensory stimuli arising caudal to an SCI (Esmail et al., 2002). The circulating levels of renin and Ang II do not change during experimentally induced AD (Mathias et al., 1981; Groothuis et al., 2010a), indicating the renin-angiotensin system does not contribute directly to the rise in blood pressure during AD. Therefore, the hypotensive action of captopril during AD could potentially be explained by a reduction in the facilitatory effect that basal levels of Ang II have on sympathetic nerve-mediated vasoconstriction.

This study tested the hypothesis that chronic SCI increases the reactivity of rat tail and mesenteric arteries to Ang II. These arteries were selected because they are known to be strongly activated during AD in rats (Laird et al., 2006; Lujan et al., 2010). Captopril reduces blood pressure in SCI rats (Osborn et al., 1989), demonstrating that Ang II contributes to the maintenance of blood pressure in this species following spinal injury. Also like in humans, the blood pressure rise during AD in rats is explained by sympathetic nerve-mediated vasoconstriction (Santajuliana et al., 1995). Furthermore, evidence indicates that SCI leads to an augmentation of sympathetic neurovascular transmission that contributes to generation of AD in both humans and rats (Teasell et al., 2000; McLachlan and Brock, 2006). Therefore, it is possible that Ang II contributes to the augmentation of nerve-evoked vasoconstriction following SCI. In both tail and mesenteric arteries, the possibilities that SCI increases the reactivity to the vascular muscle to Ang II and/or increases the facilitatory action of this peptide on sympathetic nerve-evoked constriction were investigated.

## Materials and methods

All experimental procedures were approved by the University of Melbourne Animal Experimentation Ethics Committee and they conformed to the Australian Code of Practice for the Care and Use of Animals for Scientific Purposes.

### Spinal cord transection

The spinal cord was transected in 14 female Sprague-Dawley rats (~8 weeks of age) that were anesthetized with isoflurane (2–3% in oxygen). Eight of these rats had a spinal cord transection at the T11 spinal segment and were used to study the effects of SCI on tail arteries. This lesion severs all bulbospinal connections to the preganglionic neurons that control the tail artery, which are located in the T13-L2 spinal segments (Rathner and McAllen, 1998; Smith and Gilbey, 1998). To study the effects of SCI on mesenteric arteries, the spinal cord was transected at the T4 spinal segment in six rats. In the rat, the sympathetic preganglionic neurons projecting in the splanchnic nerves are located in the spinal cord between the T4 and T12 spinal segments and transection at the T4 spinal segment will remove virtually all their bulbospinal inputs while damaging <5% of these neurons directly (Anderson et al., 1989). To lesion the spinal cord, a longitudinal incision was made through the back skin to expose the underlying musculature and the T3–4 or T10 vertebrae were identified. The muscles connecting to the spines of the vertebrae were detached and the spinal cord was exposed by a laminectomy over the T4 or T11 spinal segment (underlying the T3 and T10 vertebra, respectively; Gelderd and Chopin, 1977). The spinal cord was cut with fine scissors and the laminectomy site was closed with three layers of sutures through the adjacent vertebral musculature, the subdermal tissues, and the skin. Sterile saline (2 ml) and analgesic (0.06 mg/kg buprenorphine; Reckitt Benckiser, Sydney, NSW, Australia) were administered postoperatively by intraperitoneal injection, and the animal was placed in a cage on a heated pad to recover from the anesthesia. Additional injections of saline and analgesic were administered daily for the first 3 postoperative days. Bladders were manually expressed three times daily until the animals regained the ability to empty their own bladders. In 15 age-matched sham-operated controls, the laminectomy was performed to expose the spinal cord (7 at T4 and 8 at T11) and postoperative treatments were similar except for bladder management.

The SCI and sham-operated rats were maintained for 6–7 weeks post-surgery. The rats were killed by inhalation of CO_2_, with the concentration of CO_2_ being gradually increased. The mesentery containing the mesenteric arteries supplying the distal ~15 cm of ileum or the tail artery (2–4 cm distal to the base of the tail) were dissected and maintained in physiological saline with the following composition (in mM): 150.6 Na^+^, 4.7 K^+^, 2 Ca^+^_2_, 1.2 Mg^+^_2_, 144.1 Cl^−^, 1.3 H_2_PO^−^_4_, 16.3 HCO^−^_3_, and 7.8 glucose. This solution was gassed with 95% O_2_/5% CO_2_ and warmed to 36–37°C.

### Mechanical responses

Segments of tail artery were mounted in wire myographs and the basal conditions were normalized as described in Al Dera et al. (2012). The procedure for normalizing the basal conditions was different for mesenteric arteries. Segments of second-order mesenteric artery (~1.5 mm in length) were mounted isometrically between stainless steel wires (40 μm diameter) in two four-chamber myographs (Multi Myograph model 610M, Danish Myo Technology). To normalize the basal conditions, Laplace's equation was used to convert the measured force to the effective transmural pressure (see Mulvany and Halpern, 1977). Initially, arteries were gradually stretched to establish the inner circumference at an effective transmural pressure of 13.3 mN mm^−2^ (100 mm Hg). The lumen circumference was then adjusted to 90% of that determined at 13.3 mN mm^−2^. Using these normalization protocols, both tail arteries and mesenteric arteries were mounted at the peak of their length-force relation (Mulvany and Halpern, 1977; Yeoh et al., 2004a). For both tail arteries and mesenteric arteries the neurally and chemically evoked contractions were measured as increases in wall tension (force/2 × vessel length; Mulvany and Halpern, 1977).

After mounting, all vessels were allowed to equilibrate for at least 30 min. During this period the mesenteric arteries were treated with 1 μM capsaicin for 10 min to remove the inhibitory effects of activating the peptidergic primary afferent axons on nerve-evoked contractions (Haddock and Hill, 2011). Tail arteries are very sparsely innervated by peptidergic primary afferent axons and their activation does not inhibit nerve-evoked contractions (Li and Duckles, 1993). All vessels were then stimulated with three applications of phenylephrine (3 μM for tail arteries and 10 μM for mesenteric arteries) to confirm that the tissues were viable. The contractions to the first and second applications of phenylephrine were terminated by washout and when the third contraction had plateaued, carbachol (1 μM) was applied to confirm that the endothelium was intact.

### Ang II-induced contractions

For both mesenteric and tail arteries, non-cumulative concentration-response curves for Ang II (1–100 nM) were constructed by increasing the concentration applied by log increments. The tissues were exposed to each concentration of Ang II for 3–4 min, with the tissues washed immediately after the contractions had peaked. A 35–40 min washout period was left between applications of Ang II to minimize the effects of desensitization of Ang II receptor function.

### Electrically evoked contractions

**Figure 1:**
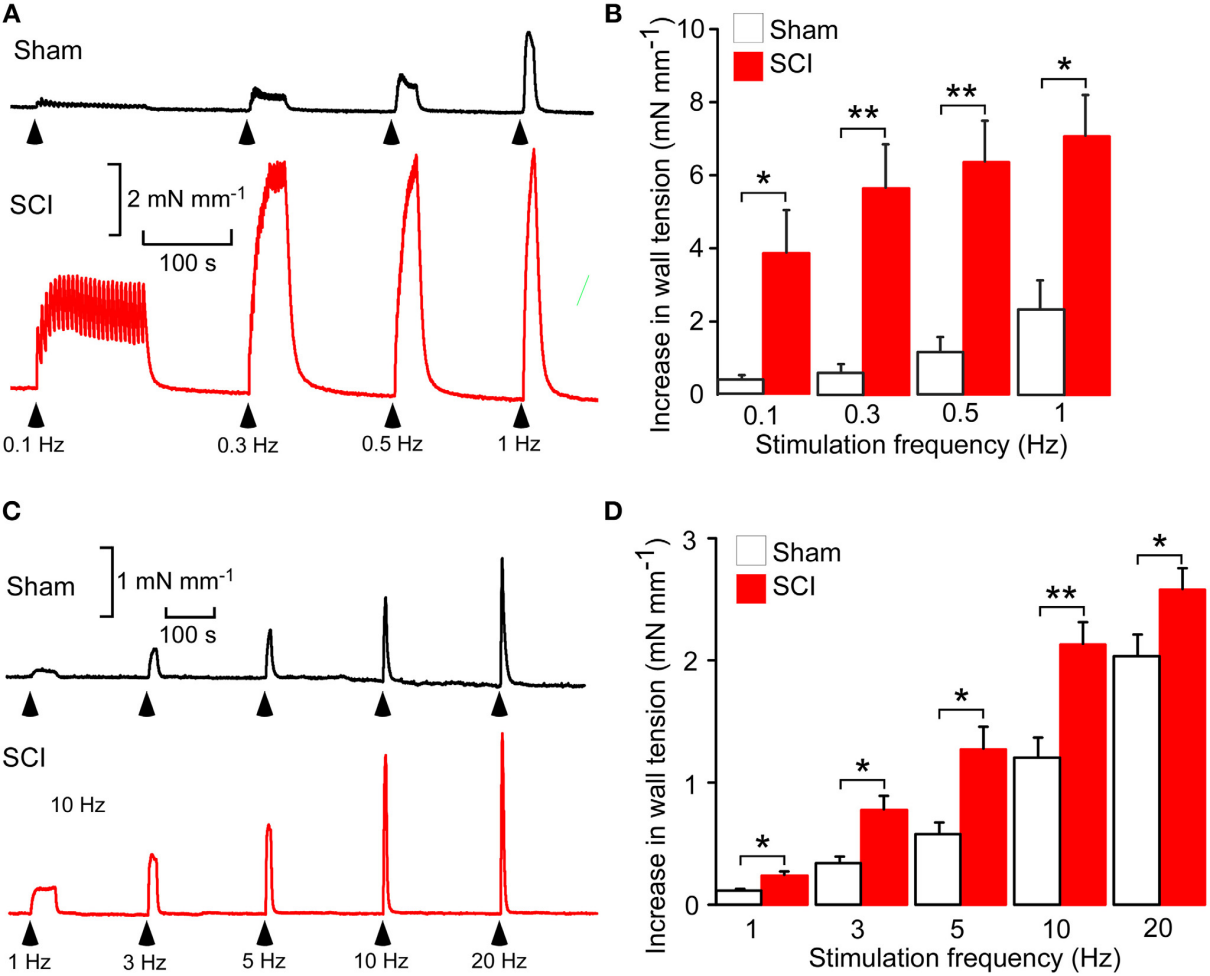
**Spinal cord injury (SCI) increased nerve-evoked contractions of both tail and mesenteric arteries. (A)** Representative traces showing contractions of a tail artery segment from a sham-operated (*black trace*) and a SCI (*red trace*) rat evoked by 25 stimuli at 0.1, 0.3, 0.5, and 1 Hz. **(B)** Peak increases in wall tension produced by these trains of stimuli in tail arteries from sham-operated (*white bars*, *n* = 8) and SCI (*red bars*, *n* = 8) rats. **(C)** Representative traces showing contractions of a mesenteric artery segment from a sham-operated (*black trace*) and a SCI (*red trace*) rat evoked by 100 stimuli at 1, 3, 5, 10, and 20 Hz. **(D)** Peak increases in wall tension produced by these trains of stimuli in mesenteric arteries from sham-operated (*white bars*, *n* = 7) and SCI (*red bars*, *n* = 6) rats. In **(B,D)**, the data are presented as a mean and SE with statistical differences between control and SCI arteries indicated by asterisks (unequal variance *t*-test; ^*^P < 0.05, ^**^P < 0.01).

Electrical stimuli (0.2 ms pulse width, 20 V for tail arteries and 15 V for mesenteric arteries) supplied by a four channel stimulator (EXP-ST-CH4, Experimetria, Balatonfüred, Hungary) were applied through platinum plate electrodes mounted either side of the tissue. It was confirmed that these stimuli produced supramaximal responses that were abolished by 0.5 μM tetrodotoxin, indicating that they were due entirely to the action of neurotransmitter released from the perivascular nerves. Initially a stimulus frequency-contraction curve was constructed for each tissue with the tail arteries being stimulated with trains of 25 stimuli at 0.1–1 Hz whereas the mesenteric arteries were stimulated with trains of 100 stimuli at 1, 3, 5, 10, and 20 Hz. Each of these trains of stimuli was separated by a 4 min interval. These different stimulus parameters were used because tail arteries were more responsive to electrical stimulation of their perivascular nerves than mesenteric arteries, which were only weakly activated by trains of stimuli at 1 Hz (cf. Figures 1A,C).

In tail arteries, the effects of Ang II (1–100 nM; Auspep, Tullamarine, VIC, Australia) on nerve-evoked contractions were investigated by assessing effects on contractions to trains of 5 stimuli at 0.5 Hz delivered at 4 min intervals. In mesenteric artery, the effects of Ang II were assessed on contractions to trains of 20 stimuli at 10 Hz delivered at 4 min intervals. Because of the potential for desensitization of Ang II receptor function, each tissue was only exposed to a single concentration of Ang II for ~16 min. The effects of losartan (selective AT_1_antagonist; Sigma-Aldrich, Castle Hill, NSW, Australia) on Ang II-induced facilitation of nerve-evoked contractions were assessed in tissues that had not previously been exposed to Ang II.

### Data analysis

The output from the myograph was recorded and analyzed using a PowerLab data acquisition system and the program Chart (ADInstruments, Bella Vista, NSW, Australia). The peak amplitudes of contractions to the trains of electrical stimuli and to phenylephrine and Ang II were measured. Relaxation responses to carbachol were expressed as a percentage reduction of the constriction to phenylephrine measured at the time carbachol was applied.

All statistical comparisons were made using SPSS 22 (IBM corporation, NY, USA). Before comparison, the data sets were checked for normality using Kolmogorov–Smirnov tests. Where data set were normally distributed, the data are presented as means ± standard error of the mean (SE) and comparisons were made with unequal variance *t*-tests (Ruxton, 2006). Where the data sets were not normally distributed, the data are presented as median and interquartile range and comparisons were made with Mann–Whitney *U*-tests. When multiple pairwise comparisons were made between data obtain in the same tissues, the *P*-values were adjusted using the false discovery rate procedure (Curran-Everett, 2000). *P*-values < 0.05 were considered to indicate significant differences. In all cases, *n* indicates the number of animals studied.

## Results

### Basal conditions and responses to phenylephrine and carbachol

After the normalization procedure the lumen diameter of tail arteries and mesenteric arteries from SCI rats did not differ significantly from those of sham-operated rats (Table 1). However, the basal wall tension after equilibration was slightly smaller (~15%) for the tail arteries from SCI rats (Table 1). The basal wall tension did not differ between mesenteric arteries from SCI and sham-operated rats (Table 1).

**Table 1:** **Basal lumen diameter and wall tension and the responses to phenylephrine (PE) and carbachol in tail and mesenteric arteries from sham-operated and SCI rats**.

	**Tail artery**			
	**Diameter (μm)**	**Basal wall tension (mN mm^−1^)**	**3 μM PE (mN mm^−1^)**	**1 μM Carbachol % Relaxation**
Sham (*n* = 8)	798 ± 26	4.5 ± 0.07	8.7 ± 0.9	62 ± 4
SCI (*n* = 8)	772 ± 25	3.9 ± 0.21	9.3 ± 1.1	51 ± 6
*P*	0.50	<0.05^*^	0.52	0.18
	**Mesenteric artery**			
	**Diameter (μm)**	**Basal wall tension (mN mm^−1^)**	**10 μM PE (mN mm^−1^)**	**1 μM Carbachol % Relaxation**
Sham (*n* = 7)	296 ± 10	1.3 ± 0.1	4.5 ± 0.2	70 ± 10
SCI (*n* = 6)	290 ± 10	1.3 ± 0.1	4.5 ± 0.2	76 ± 5
*P*	0.87	0.97	0.87	0.64

*Data are present as mean ± SE and comparisons between arteries from sham-operated and SCI rats were made with unequal variance t-tests*.

* *indicates significant differences*.

For both tail arteries and mesenteric arteries, the peak increase in wall tension produced by phenylephrine (3 μM for tail artery and 10 μM for mesenteric artery) did not differ between vessels from SCI and sham-operated rats (Table 1). The % relaxation produced by carbachol of phenylephrine constricted tail arteries and mesenteric arteries also did not differ between vessels from SCI and sham-operated rats (Table 1).

### SCI increased nerve-evoked contractions

As previously reported (Yeoh et al., 2004a; Brock et al., 2006; Al Dera et al., 2012), in both tail and mesenteric arteries SCI increased the size of nerve-evoked contractions (Figure 1). However, the magnitude of this effect was greater in tail arteries than in mesenteric arteries, with contractions to trains of stimuli at 1 Hz being increase by ~2-fold in mesenteric arteries and ~3-fold in tail arteries.

### SCI increased the reactivity of tail arteries to ang II

**Figure 2:**
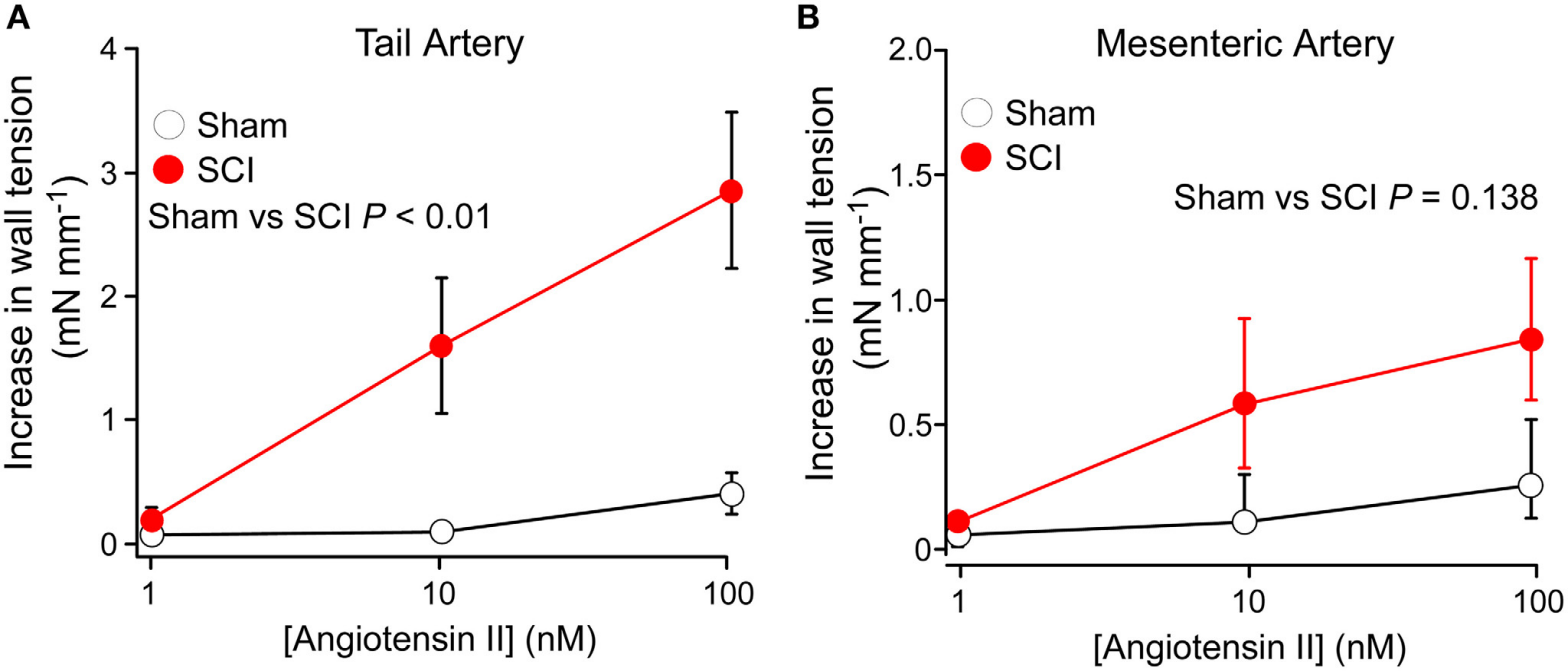
**Spinal cord injury (SCI) increased the responsiveness of tail arteries to angiotensin II (Ang II). (A,B)** Concentration–contraction curves for Ang II (1–100 nM) in tail arteries **(A)** and mesenteric arteries **(B)** from sham-operated (*open circles*; tail artery *n* = 8, mesenteric artery *n* = 7) and SCI rats (*solid red circles*; tail artery *n* = 8, mesenteric artery *n* = 6). In **(A)**, the data are presented as mean and SE and statistical comparisons between the summed data for sham-operated and SCI arteries was made with a unequal variance *t*-test (*P* indicated in **A**). In **(B)**, the data are present as median and interquartile range and statistical comparison between the summed data for sham-operated and SCI arteries groups was made with an Mann–Whitney *U*-test (*P* indicated in **B**).

Figure 2 shows concentration-contraction curves for Ang II (1–100 nM) in tail arteries (Figure 2A) and mesenteric arteries (Figure 2B) from sham-operated and SCI rats. In tail arteries from SCI rats (*n* = 8), the responses to Ang II were more variable but they were significant larger than those from sham-operated rats (*n* = 8; Figure 2A). In mesenteric arteries from both control and SCI rats, the contractions to Ang II at 10 and 100 nM were small and variable but there was a tendancy for the Ang II-evoked contractions of mesenteric arteries from SCI rats (*n* = 6) to be larger than those from sham-operated rats (*n* = 7; Figure 2B). This difference did to reach the level of statistical significance (Figure 2B). However, because of the considerable variability of the responses to Ang II, a larger number of control and SCI arteries would need to be tested to exclude the possibilty that SCI increases the sensitivity of mesenteric arteries to Ang II.

### Ang II increased nerve-evoked contractions

**Figure 3:**
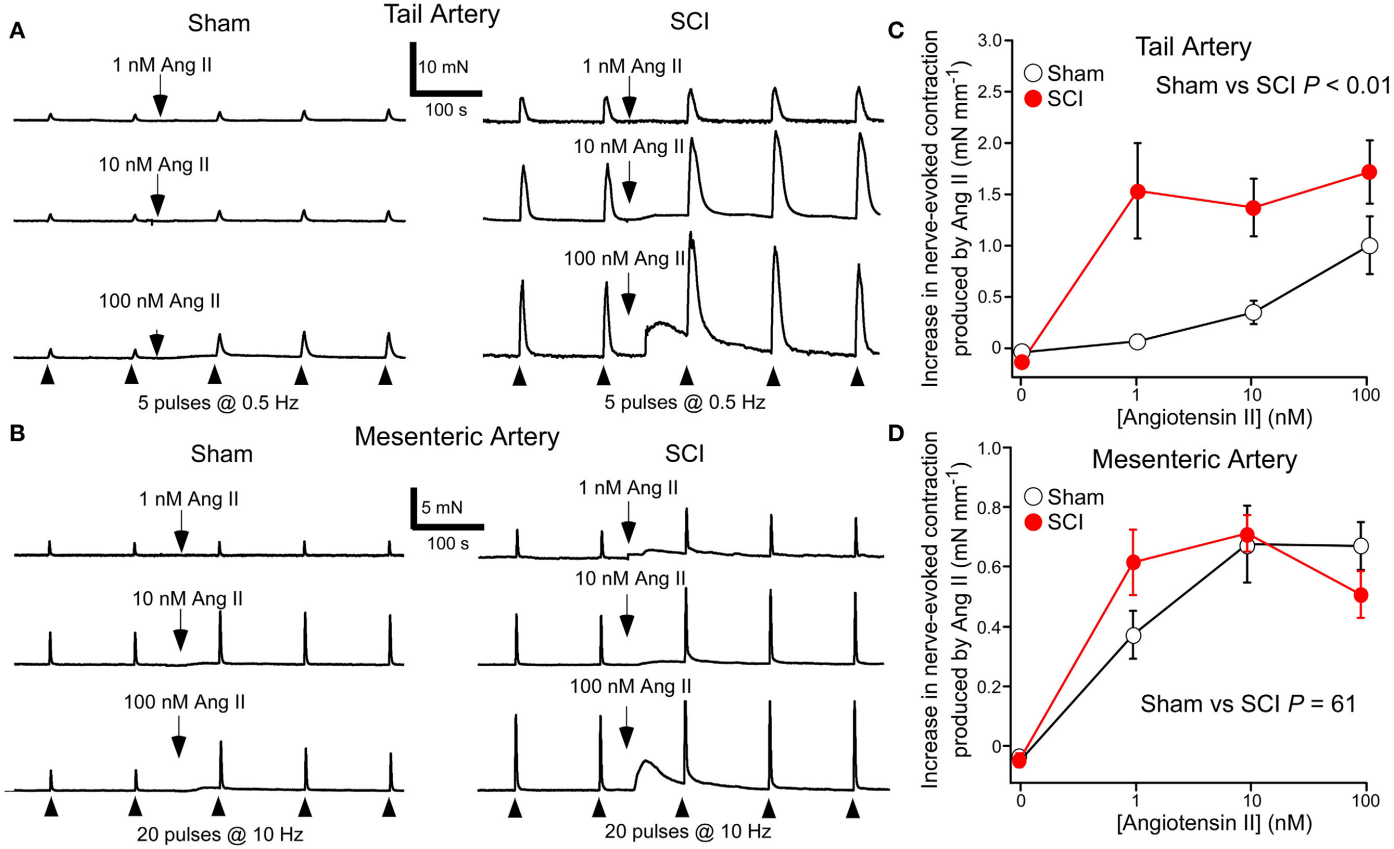
**Angiotensin II (Ang II) increased nerve-evoked contractions of both tail arteries and mesenteric arteries**. In tail arteries, but not mesenteric arteries, the increment in the size of nerve-evoked contractions produced by Ang II was markedly increased by spinal cord injury (SCI). **(A)** Representative traces showing contractions of tail artery segments from a sham-operated (*left panel*) and a SCI (*right panel*) rat evoked by 5 stimuli at 0.5 Hz before and during application of 1, 10, and 100 nM Ang II. **(B)** Representative traces showing contractions of mesenteric artery segments from a sham-operated (*left panel*) and a SCI (*right panel*) rat evoked by 20 stimuli at 10 Hz before and during application of 1, 10, and 100 nM Ang II. **(C,D)** The increment in the size of nerve-evoked contractions produced by Ang II (1–100 nM) in tail arteries **(C)** and mesenteric arteries **(D)** from sham-operated (*open circles*; tail artery *n* = 8, mesenteric artery *n* = 7) and SCI (*solid red circles*; tail artery *n* = 8, mesenteric artery *n* = 6) rats. The “zero” Ang II data is for time-matched controls with no Ang II added. In **(C,D)**, the data are present as mean and SE and statistical comparisons between the curves were made by comparing the summed data for sham-operated and SCI arteries using unequal variance *t*-tests (*P* indicated in **C**,**D**).

In tail arteries (Figure 3A) and mesenteric arteries (Figure 3B), the contraction to Ang II peaked within 4 min of application and then rapidly declined. In contrast, the facilitatory effect of Ang II on nerve-evoked contractions peaked within 4–8 min of application and then slowly declined in some tissues. For this reason the effects of Ang II on nerve-evoked contractions were measured at the second response in its presence (~7 min after adding Ang II).

At 1 nM, Ang II increased nerve-evoked contractions of tail arteries from SCI rats and this facilitatory action of Ang II did not increase when 10 or 100 nM was applied (Figure 3C). By contrast, in tail arteries from sham-operated rats, the facilitatory action of Ang II on nerve-evoked contractions increased with the concentration applied (1–100 nM), with 1 nM having little effect on these responses (Figure 3C). Ang II also increased the amplitude of nerve-evoked contractions of mesenteric arteries (Figure 3B), but at 1–100 nM the increment in wall tension produced by this agent did not differ between the vessels from sham-operated and SCI rats (Figure 3D).

### The facilitatory effect of ang II on nerve-evoked contractions was reduced by losartan

**Figure 4:**
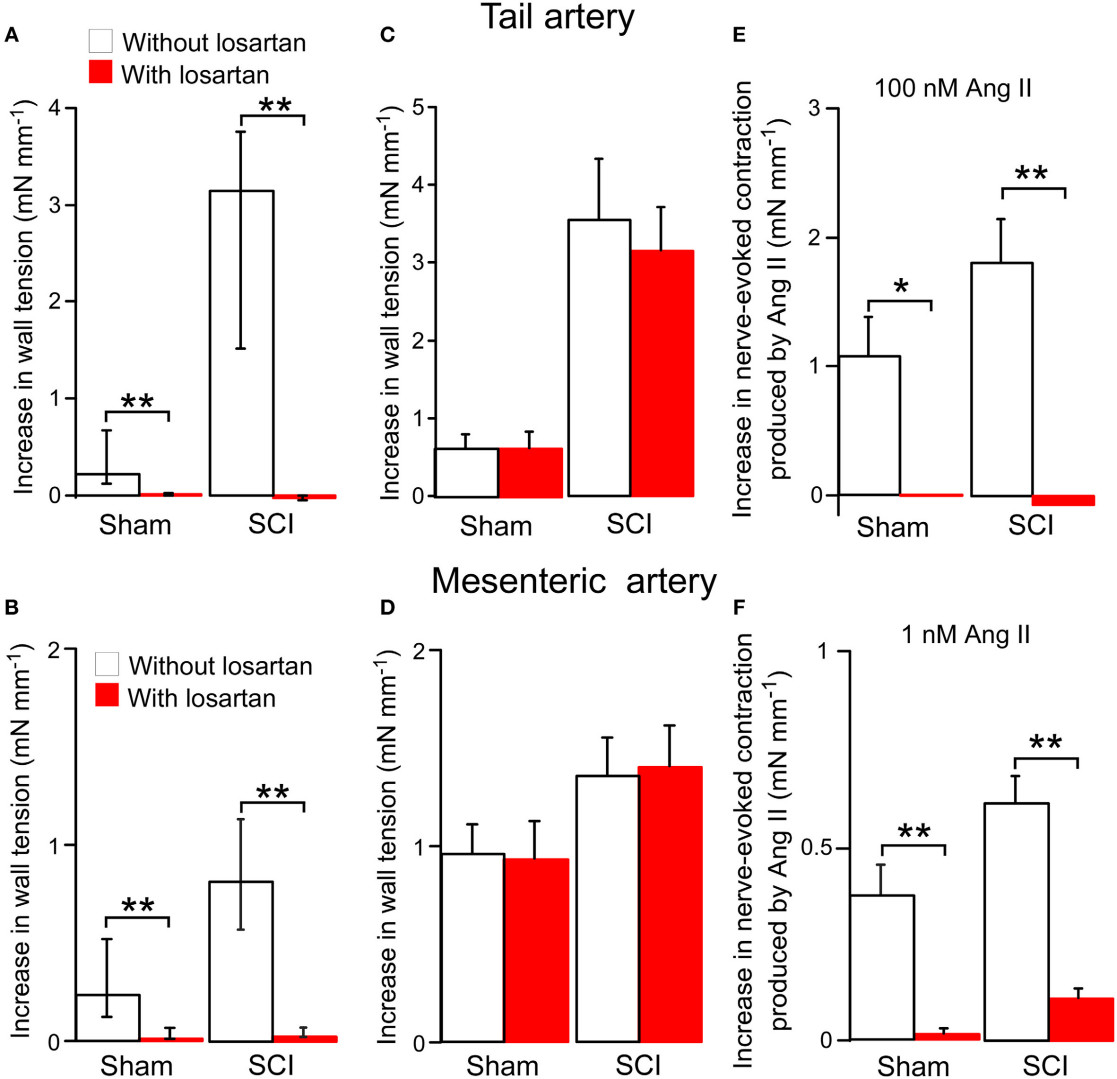
**The angiotensin II (Ang II) type 1 receptor antagonist losartan (100 nM) blocked both Ang II-evoked contractions and the enhancement of nerve-evoked contractions produced by Ang II in tail arteries and mesenteric arteries from sham-operated and spinal cord injured (SCI) rats**. On its own losartan did not change nerve-evoked contractions. **(A,B)** Peak increases in wall tension produced by 100 nM Ang II in tail arteries (**A**; sham-operated *n* = 8, SCI *n* = 8) or mesenteric arteries (**B**; sham-operated *n* = 7, SCI *n* = 6) in the absence (*white bars*) or in the presence (*red bar*) of losartan. **(C,D)** Peak increases in wall tension evoked by nerve stimulation (tail artery: 5 stimuli at 0.5 Hz; mesenteric artery: 20 stimuli at 10 Hz) before (*white bar*) and during (*red bar*) application of losartan in tail arteries (**C**; sham-operated *n* = 6, SCI *n* = 6) and mesenteric arteries (**D**; sham-operated *n* = 7, SCI *n* = 6). **(E)** The increment in the size of nerve-evoked contractions produced by 100 nM Ang II in tail arteries in the absence (*white bars*; sham-operated *n* = 7, SCI *n* = 7) or in the presence (*red bars*; sham-operated *n* = 7, SCI *n* = 7) of losartan. **(F)** The increment in the size of nerve-evoked contrations produced by 1 nM Ang II in mesenteric arteries (sham-operated *n* = 7, SCI *n* = 6) in the absence (*white bars*) or in the presence (*red bars*) of losartan. Data are presented as median and interquartile in **(A,B**) and mean and SE in **(C–F)**. Statistical differences between responses in the absence and in the presence of losartan are indicated by asterisks (by Mann–Whitney *U*-tests (in **A,B**) or unequal variance *t*-tests (in **E,F**); ^*^P < 0.05, ^**^P < 0.01).

The effects of the Ang II type 1 (AT_1_) receptor antagonist losartan on responses to Ang II were investigated. In tail arteries and mesenteric arteries from both sham-operated and SCI rats, 100 nM losartan abolished contractions to 100 nM Ang II (Figures 4A,B). At this concentration, losartan on its own had no effect on nerve-evoked contractions of either artery type (Figures 4C,D). In tail arteries from both groups of rats, 100 nM losartan abolished the augmentation of nerve-evoked contractions produced by 100 nM Ang II (Figure 4E). Similarly, in mesenteric arteries from both groups of rats, the facilitatory action of 1 nM Ang II on nerve-evoked contraction was greatly reduced by this concentration of losartan (Figure 4F).

## Discussion

The main finding of this study is that chronic SCI has differential effects on the reactivity of tail and mesenteric arteries to Ang II. In tail arteries, but not mesenteric arteries, SCI produced a large increase in reactivity of the vascular muscle to Ang II and also markedly increased the facilitation of nerve-evoked contractions produced by Ang II. Therefore, the hypothesis that SCI produces hyperreactivity of the vaculature to Ang II is supported but the changes detected depend on the vessel studied.

The increase in nerve-evoked contraction produced by SCI is almost certainly explained by the decrease in ongoing sympathetic nerve activity caudal to the lesion (Maiorov et al., 1997; Yeoh et al., 2004b). Short-term abolition of sympathetic neuroeffector transmission in smooth muscles by decentralization (by lesioning the preganglionic inputs to their supplying postganglionic neurons) or by denervation increases their sensitivity to α-adrenoceptor agonists (Fleming and Westfall, 1988). Accordingly, decentralization, denervation and SCI produced a transient increase in sensitivity of tail artery vascular muscle to α_1_-adrenoceptor agonists within 1–2 weeks of surgery but this had subsided by 6–8 weeks (Yeoh et al., 2004a,b; Tripovic et al., 2010). While denervated tail arteries did not have maintained hyperreactivity to α_1_-adrenoceptor agonists, these vessels had a marked and maintained increase in their reactivity to Ang II (Tripovic et al., 2010). A similar increase in reactivity to Ang II has been demonstrated in decentralized tail arteries 2 weeks post-operatively (Tripovic et al., 2010) and here SCI for 6–7 weeks increased reactivity to Ang II. Therefore, in tail artery, chronic interruption of sympathetic neuroeffector transmission increases the reactivity of the vascular muscle to Ang II.

The long-term effects of surgically induced denervation on the reactivity of mesenteric arteries have not been reported. However, it has been reported that SCI for 7 weeks does not increase sensitivity of the vascular muscle in mesenteric arteries to α_1_-adrenoceptor agonists (Brock et al., 2006). In the present study, the highest concentration of Ang II tested (100 nM) produced a small contraction of mesenteric arteries from sham-operated rats (~5% of the contraction to 10 μM phenylephrine). In SCI mesenteric arteries, there was a tendency for the contractions to 100 nM Ang II to be larger (~15% of the contraction to 10 μM phenylephrine). However, the relatively large variability of the contractions to Ang II observed in mesenteric arteries means that a larger number of vessels would need to be studied to establish whether or not SCI increases vascular muscle reactivity to this peptide. Irrespective of the effects of SCI on Ang II-evoked contractions of mesenteric arteries, the facilitatory effect of this agent on nerve-evoked contractions of these vessels was not significantly changed by SCI.

The present study did not investigate the mechanisms underlying the facilitation of nerve-evoked contractions produced by Ang II. In both tail and mesenteric arteries, Ang II has been demonstrated to increase noradrenaline release from the perivascular nerve terminals but the maximally effective concentration in tail arteries (~1 μM; Cox et al., 1996b) is about 100-fold greater than that in mesenteric arteries (~10 nM; Morato et al., 2006). In mesenteric arteries, 10 nM Ang II increased nerve-evoked contractions of mesenteric arteries but did not increase contractions to exogenously applied noradrenaline (Balt et al., 2001). Therefore, in mesenteric arteries, the facilitatory effect of Ang II on nerve-evoked contractions appears to be explained by an increase in neurotransmitter release from the sympathetic nerve terminals. As the facilitatory effect of Ang II on nerve-evoked contractions did not differ between mesenteric arteries from SCI and sham-operated rats, we assume that the facilitatory effect of Ang II on noradrenaline release is not changed by SCI.

In tail arteries, Ang II at 100 nM (the highest concentration used in the present study) increased contractions to both nerve stimulation and exogenously applied noradrenaline but did not increase noradrenaline release (Thorin and Atkinson, 1994). Thus, in arteries from sham-operated rats, the augmentation of nerve-evoked contractions produced by Ang II is most likely due to a postjunctional action of this peptide. As SCI markedly increased contractions of tail arteries produced by Ang II, a postjunctional increase in smooth muscle reactivity is likely to contribute the augmented facilitatory effect of this peptide on nerve-evoked contractions. However, the possibility that SCI increases the sensitivity of the sympathetic nerve terminals to Ang II in tail artery cannot be excluded. As previously reported (Cox et al., 1996a; Balt et al., 2001), the AT_1_ receptor antagonist losartan blocked both Ang II-induced contractions and the augmentation of nerve-evoked contractions produced by this peptide in both tail and mesenteric arteries. As AT_1_ receptors mediate both the prejunctional and postjunctional actions of Ang II in these arteries (Cox et al., 1995; Ziogas and Vessey, 2001), these findings do not resolve the site at which Ang II acts to increase nerve-evoked contractions.

Groothuis et al. (2010b) have provided evidence that suggests arterial reactivity to Ang II is increased in the legs of patients with an SCI in the middle to lower thoracic region (T4–T12) of the spinal cord. When supine these SCI subjects had a higher arterial vascular resistance in the legs than did able-bodied controls. By contrast, in the forearms where neural control the vasculature is unaffected by the SCI, arterial vascular resistance did not differ between the SCI and control groups. Under these conditions, the AT_1_ receptor antagonist irbesartan reduced arterial vascular resistance in the legs of SCI subjects but not in those of control subjects. Irbesartan had no effects on arterial vascular resistance in the forearms of both groups. As it would be predicted that the vasculature in the arms and legs is exposed to the same circulating concentration of Ang II, these findings suggest the vasculature in the legs of the SCI subjects is hyperreactive to Ang II. In accord with this suggestion, the dose of intravenously infused Ang II required to raise blood pressure by 20 mmHg was reduced in quadriplegic SCI patients (Krum et al., 1992).

The concentrations of Ang II that constrict animal and human arterial vessels *in vitro* are typically much higher than those in blood (Camarda et al., 2002). In humans, the normal levels of Ang II in plasma are in the range 1–10 pM (Hermann et al., 1988) and in rats is ~15 pM (Hermann et al., 1988). In the study of Groothuis et al. (2010b), patients with middle to lower thoracic SCI had slightly but significantly higher levels of circulating Ang II (~5 pM) than did able bodied subjects (~3 pM). The low level of Ang II in blood raises the question as to the concentration that is present in the vascular wall. Ang II can be generated locally in vascular wall by conversion of angiotensin I (Ang I) to Ang II (Schuijt et al., 2002). This has been demonstrated in both rat tail and mesenteric arteries (Collis and Keddie, 1981; Ziogas et al., 1984). The circulating levels of Ang I are higher than those of Ang II (~20 pM in humans and ~70 pM in rats; Hermann et al., 1988) and it has been estimated that the concentration of Ang II generated from Ang I in the interstitial fluid within the vascular wall may be up to 10-fold higher than the circulating level of Ang I (Schuijt et al., 2002). In addition, some blood vessels have been demonstrated to synthesize angiotensinogen that is converted locally, by renin taken up from the circulation, to Ang I that is then converted to Ang II (Bader et al., 2001). For these reasons it is possible that the concentration of Ang II present in the vascular wall is much higher than the circulating level. It is also possible that the increased contribution of Ang II to maintenance of vascular resistance in legs of SCI subjects is explained by increased local synthesis of Ang II, rather than an increase in vascular reactivity to this peptide (Groothuis et al., 2010b). In the present study, the SCI-induced enhancement of nerve-evoked contractions cannot be attributed to endogenously synthesized Ang II as it was not reduced by the AT_1_-receptor receptor antagonist losartan (Figures 4C,D).

### Significance for cardiovascular consequences of SCI

This is the first study to directly demonstrate that SCI increases the reactivity of arterial vessels to Ang II. This change potentially contributes to restoration of blood pressure control following SCI in both human and rats. In both humans and rats, the splanchnic vascular bed is believed to play a dominant role in the rise in blood pressure during AD (Mathias and Frankel, 1999; Lujan et al., 2010). The present study demonstrates that Ang II amplifies nerve-evoked constrictions of mesenteric arteries but that the magnitude of this effect was not changed by SCI. So while blockade of Ang II actions will potentially reduce nerve-evoked vasoconstriction of arterial vessels in splanchnic vascular bed, it would not be expected to reduce the SCI-induced hyperreactivity of these vessels to neural activation. However, while captopril has been reported to be effective in reducing blood pressure during AD in SCI patients (Esmail et al., 2002), at present it is unknown whether this treatment is effective during AD in rats.

The tail artery controls the blood flow to tail skin, which is reduced during AD evoked by colorectal distension in SCI rats (Laird et al., 2006). Neural regulation of skin blood flow can potentially contribute to the regulation of peripheral resistance (see Rowell, 1984). In humans, during AD there is widespread cutaneous vasoconstriction with cold skin being a characteristic feature of this condition (Mathias and Frankel, 1999). Therefore, concurrent activation of arterial vessels supplying skin, muscle, and visceral organs almost certainly contributes to the overall increase in peripheral resistance during AD. Thus, it is possible that the blood pressure lowering effect of captopril during AD is due, in part, to a reduction in hyperreactivity of cutaneous arterial vessels to neural activation. It is also possible that the influence of Ang II on neural activation of arterial vessels supplying other vascular beds (e.g., skeletal muscle, kidney) is increased by SCI.

SCI-induced changes in the neural regulation of skin blood flow are believed to contribute to both the etiology of pressure sores and the slowing of skin wound repair in SCI patients (Teasell et al., 2000). In patients with SCI, it is possible that hyperreactivity of the cutaneous vasculature to Ang II contributes to both the reduction in ischemia-induced vasodilation that occurs following removal of a pressure stimulus applied to skin (Schubert and Fagrell, 1991) and to impairing axon reflex-evoked vasodilation in skin (Kuesgen et al., 2002). Both of these changes contribute to impairing skin function in SCI patients (Teasell et al., 2000).

In summary, SCI changes the reactivity of the tail artery to Ang II. This effect may contribute to modifying the contribution of Ang II to vascular control following SCI. In addition, as the tail artery supplies blood to tail skin, it is possible that increased reactivity of cutaneous arterial vessels to Ang II contributes to impairing skin function following SCI. These possibilities require further investgation.

### Conflict of interest statement

The authors declare that the research was conducted in the absence of any commercial or financial relationships that could be construed as a potential conflict of interest.
